# The Book World of Medicine and Science

**Published:** 1893-03-11

**Authors:** 


### The Book World of Medicine and Science.

### TO OUR READERS.

We propose in future to give more prominence to books and literary matters. The great popularity which The Hospital has attained amongst members of the profession, and the increasing desire manifested to have early intelligence of all medical and scientific books of value or interest, have led us to make arrangements for supplying this information. Any readers who are specially interested in books are invited to write to the Editor, 428, Strand, W.C., and to send from time to time their impressions and criticisms of new books, as well as items of literary intelligence which may be calculated to interest or amuse. There are many literary men and women amongst the readers of The Hospital, and with their co-operation and that of the publishers, we hope to make "The Book World of Medicine and Science" one of the most complete and attractive features of the paper. Co-operation and help are therefore invited, and the more promptly one or both come to hand the better we shall be pleased.

### FIRST IMPRESSIONS OF BOOKS RECEIVED.

It is requested that all books intended to be noticed in this column may be sent to the Editor, at the Office, 428, Strand, W.C., not later than Tuesday evening in each week.

A Pocket Medical Dictionary. By George M. Gould, A.M., M.D., author of "A New Medical Dictionary." (London: H. K. Lewis.)

A true *multum in parvo*. Contains 12,000 words, and gives their correct spelling, pronunciation, and meaning. There are also some useful anatomical and pathological tables, and at the end a table of doses. All this in a form and size easily carried in the coat pocket.

Mental Nursing, or Lectures for Asylum Attendants. By William Harding, M.B. (Ed.), M.R.C.P. (Lond.), Assistant Medical Officer, Female Department, Berrywood, Northampton. (London: The Scientific Press, Limited, 1893.)

A special book for nurses. It gives in short compass and plain words a simple account of the anatomy and physiology of the nervous system and of the various forms of insanity. Other chapters are devoted to a description of the special duties of attendants on the insane.

The Medical Annual and Practitioner's Index, 1893. Eleventh year. (Bristol: John Wright and Co.)

We are glad to welcome the new volume of this excellent annual. It covers a very wide field, and the articles are concise but not scrappy, and together they make up an invaluable book of reference.

A Manual of Bacteriology. By A. B. Griffiths, Ph.D., F.R.S.E., F.C.S. (London: William Heinemann.)

This is one of Heinemann's Scientific Series, which are intended to impart such a knowledge of the practical sciences as every educated man ought to possess. They are not intended for specialists. This volume deals with the most interesting and practically important advance of science made within the last twenty years. Dr. Griffiths' work is good, and he gives abundant references for those who wish to pursue the study further.

**Sheffield Medical Journal.**

THE BOOK WORLD OF MEDICINE AND SCIENCE.—(*continued*).

Various Forms of Hysterical or Functional Paralysis. By H. Charlton Bastian, M.A., M.D., F.R.S. (London: H. K. Lewis.)

The most scientific and careful attempt yet made to elucidate a very important and difficult subject. Dr. Bastian deals with it in a masterly way, and his scientific acumen is blended with great literary ability.

Essays upon Heredity and Kindred Biological Problems. By Dr. August Weismann, Professor in the University of Freiburg, in Breslau. Edited by E. B. Poulton, M.A., F.R.S., Selmar Schönland, Ph.D., and A. E. Shipley, M.A. Second Edition. (Oxford: At the Clarendon Press.)

The second edition appears in two volumes. The first volume is a reprint of the former edition, with a few additions and emendations. The second volume consists of four additional essays, in which Weismann develops his views more completely. It will be welcomed by all thoughtful biologists and philosophic thinkers.

The Sheffield Medical Journal. January, 1893.

This number contains an address by Dr. Pye Smith, papers on Chloroform and Ether as Anæsthetics, Notes of interesting cases, Abstracts and Reviews.

The Provincial Medical Journal. Edited by Thomas M. Dolan, M.D. February, 1893.

There is a good portrait and very brief sketch of Professor Schäfer, especially acceptable to his numerous old pupils. Then follows a long article on the treatment of Hip Disease by Thomas's method, written by Drs. Jones and Pridlon—illustrations numerous, but rough. Dr. Joseph Coats contributes an admirable paper on the Infective Nature of Cancer, in which he advances the arguments for and against the microbic theory of this disease, and advocates the maintenance of an open mind on the point.

### PRACTITIONERS' BOOKSHELF.

This dissertation was written as a thesis for the degree of doctor of medicine of the University of London under the new regulations. It is largely the outcome of wide and careful study of the writings of other physicians, but it contains also some good original matter. Dr. Forsbrook has made careful investigation of the blood in several cases, and in all he has found a notable diminution of the oxygen carrying homoglobin. He is inclined to attribute the disease to this initial fact—the want of oxygen telling upon the vaso-motor centres of the joints, muscles, and nerves. Special interest attaches to any reasonable theory of the causation of disease when founded upon well-attested facts. But we imagine that Dr. Forsbrook himself would admit that his facts have not proved his case, and that his pathological suggestion is hardly more at present than a plausible theory. Further investigation will be needed to establish and perhaps to confute it. It is a very interesting record that is told in the chapter devoted to the history of this disease. It is certainly of great antiquity, for signs are found in bones dating back 1300 years B.C. The gradual separation of the disease from rheumatism and gout, the evolution of the theories of its causation and the ever-extending knowledge of the phenomenon of the disease which is now known to be by no means limited to the changes in the joints, makes the historical chapter of Dr. Forsbrook's essay quite one of the most interesting.

"A Dissertation on Osteo-Arthritis." By W. H. Russell Forsbrook, M.D.Lond., M.R.C.S., Consulting Medical Officer to the Government of the Cape of Good Hope, formerly Surgical Registrar to Westminster Hospital. (London: H. K. Lewis, 1893.)

